# Lysosomal adaptation: How cells respond to lysosomotropic compounds

**DOI:** 10.1371/journal.pone.0173771

**Published:** 2017-03-16

**Authors:** Shuyan Lu, Tae Sung, Nianwei Lin, Robert T. Abraham, Bart A. Jessen

**Affiliations:** 1 Drug Safety Research and Development, Pfizer Inc., San Diego, CA, United States of America; 2 iXCells Biotechnologies, San Diego, CA, United States of America; 3 Oncology Research Unit, Pfizer Inc., San Diego, CA, United States of America; Niigata Daigaku, JAPAN

## Abstract

Lysosomes are acidic organelles essential for degradation and cellular homoeostasis and recently lysosomes have been shown as signaling hub to respond to the intra and extracellular changes (e.g. amino acid availability). Compounds including pharmaceutical drugs that are basic and lipophilic will become sequestered inside lysosomes (lysosomotropic). How cells respond to the lysosomal stress associated with lysosomotropism is not well characterized. Our goal is to assess the lysosomal changes and identify the signaling pathways that involve in the lysosomal changes. Eight chemically diverse lysosomotropic drugs from different therapeutic areas were subjected to the evaluation using the human adult retinal pigmented epithelium cell line, ARPE-19. All lysosomotropic drugs tested triggered lysosomal activation demonstrated by increased lysosotracker red (LTR) and lysosensor green staining, increased cathepsin activity, and increased LAMP2 staining. However, tested lysosomotropic drugs also prompted lysosomal dysfunction exemplified by intracellular and extracellular substrate accumulation including phospholipid, SQSTM1/p62, GAPDH (Glyceraldehyde 3-phosphate dehydrogenase) and opsin. Lysosomal activation observed was likely attributed to lysosomal dysfunction, leading to compensatory responses including nuclear translocation of transcriptional factors TFEB, TFE3 and MITF. The adaptive changes are protective to the cells under lysosomal stress. Mechanistic studies implicate calcium and mTORC1 modulation involvement in the adaptive changes. These results indicate that lysosomotropic compounds could evoke a compensatory lysosomal biogenic response but with the ultimate consequence of lysosomal functional impairment. This work also highlights a pathway of response to lysosomal stress and evidences the role of TFEB, TFE3 and MITF in the stress response.

## Introduction

Lysosomes are single membrane-enclosed compartments filled with acid hydrolytic enzymes (e.g. cathepsins) that digest macromolecules and organelles from both external and internal origins via endocytosis, phagocytosis, and autophagy degradation pathways. For optimal activity of the acid hydrolases, lysosomes must maintain a low internal pH of about 4–5. Recent compelling evidence has indicated that lysosomes play a critical role in nutrient sensing and signaling pathways involved in cell metabolism and growth. For instance, the kinase complex mammalian target of rapamycin complex 1 (mTORC1), a master controller of cell and organism growth, needs to be translocated onto the lysosomal surface to exert its activity [1]. Lysosomal dysfunctions not only associate with various genetic lysosomal storage diseases (LSDs), but also play a role in neurodegenerative diseases such as Alzheimer’s disease [2, 3]. In addition, a close relationship between autophagy, lysosomal function and cellular aging has been established [4].

Until recently, lysosomes had been considered inert organelles with simple degradation functions. However, the discovery that lysosomal genes and functions are differentially regulated at the transcriptional level under stress conditions indicates that lysosomes adapt to different intracellular and extracellular cues [5–7]. The coordinated lysosomal enhancement and regulation (CLEAR) network was identified as controlling multiple genes involved in lysosomal biogenesis and lysosome-related functions, including autophagy and endocytosis [5, 8]. Four basic helix-loop-helix transcription factors (Microphthalmia-associated transcription factor (MITF), Transcription Factor EB (TFEB), Transcription Factor E3 (TFE3) and Transcription Factor EC (TFEC)) that belong to the MiT superfamily can bind CLEAR sites in the promotor region of the target genes. Under normal conditions, TFEB and TFE3 are located in the cytoplasm, whereas during starvation or chloroquine-induced lysosomal stress, TFEB and TFE3 rapidly translocate to the nucleus to initiate lysosomal biogenesis [9, 10]. mTORC1 activity is critical in modulating the phosphorylation status of TFEB and TFE3 and subsequently their cellular location. The mTORC1-TFEB/TFE3 axis underlines the lysosome-to-nucleus signaling mechanism, equipping lysosomes with the ability to adapt to various cues, including lysosomal changes.

For many decades, it has been known that weakly basic lipophilic compounds can accumulate in acidic organelles, including lysosomes. Noble laureate and discoverer of lysosomes, Christian de Duve and his colleagues wrote an elegant commentary discussing the concept of lysosomotropism, a mechanism for accumulation [11]. The pH gradient between the lysosomal lumen (≈ 4.5) and the cytosol (≈ 7.4) drives hyper-accumulation of basic lipophilic compounds via pH partitioning. Generally, the lipophilic free bases are believed to easily traverse lipid bilayers, becoming trapped in the acidic environment of the lysosome due to ionization, which decreases the permeability of the compound. Large amounts of basic lipophilic compounds can accumulate in lysosomes. For instance, chloroquine, a well-known lysosomotropic compound, can easily reach a concentration in excess of 20 mM inside lysosomes yielding a ratio several hundred-fold higher than outside of the cells [11]. Two physicochemical properties, basic pKa (acid dissociation constant for the conjugated acid of the weak base) and clogP (partition coefficient between octanol and water, representing membrane permeability) affect the drug accumulation by influencing the extent of lysosomal trapping and regulating the kinetics of passive permeation, respectively.

It has been shown that lysosomotropic compounds can increase lysosomal pH demonstrated by decrease of LTR staining after compound sequestration which could lead to suboptimal conditions for lysosomal digestion. However LTR increase was also reported post lysosomotropic compounds indicating pH recovery after compound sequestration [12, 13]. Kinetic nature of lysosomal change with lysosomotropic compounds need to be further addressed and more importantly how cells respond to lysosomotropic compounds requires additional investigation. In the current study we demonstrated the dynamic change of lysosomes with surprising lysosomal activation after 4 hrs of lysosomotropic compounds treatment. The involvement of mTOR and calcium signaling in the lysosomal response to the lysosomotropic drugs was also established. These studies reveal the pathways of cellular response to lysosomal stress induced by lysosomotropic compounds. Although lysosomal functional restoration is still perturbed, this adaptive response appears protective towards cytotoxicity.

## Results

### Lysosomal pH modulation

The purpose of these studies was to characterize the cellular impact of lysosomotropic compounds. As shown in Table 1, eight therapeutically and structurally diverse drugs, including chloroquine, fluoxetine, imipramine, dimebon, tamoxifen, chloropromazine, amitriptyline, and verapamil were selected. The physicochemical properties including clogP and basic pKa are listed for all compounds and they all carry a basic moiety with basic pKas ranging from 8.68 to 10.47 and are lipophilic with clogP spanning from 3.49 to 6.24.

**Table 1:** Physicochemical properties, impact on autophagy and concentrations used in the studies.

Compound Name	clogP	Basic pKa	Drug Class	Autophagy	Concentrations(μM)
Chloroquine	5.06	10.47	Antimalarial	Inhibitor [41]	100, 50, 25
Fluoxetine	4.57	10.06	Serotonin Selective Re-uptake inhibitors (Antidepressants)	Enhancer [42]	100, 50, 25
Imipramine	5.04	9.49	Tricyclic Antidepressants	Enhancer [43]	200, 100, 50
Latrepirdine	3.49	9.05	Antihistamine	Enhancer [44, 45]	200. 100. 50
Tamoxifen	6.24	8.68	Estrogen Receptor Antagonist	enhancer[46]	50, 25, 12.5
Chloropromazine	5.3	9.41	Antipsychotic	Enhancer [47]	50, 25, 12.5
Amitriptyline	4.41	9.18	Tricyclic Antidepressants	Enhancer [48]	200, 100, 50
Verapamil	4.02	8.97	L-Type Calcium Channel Blocker	Enhancer [49]	200, 100, 50

Note: physicochemical properties were determined using ACD/Labs software.

**Fig 1:**
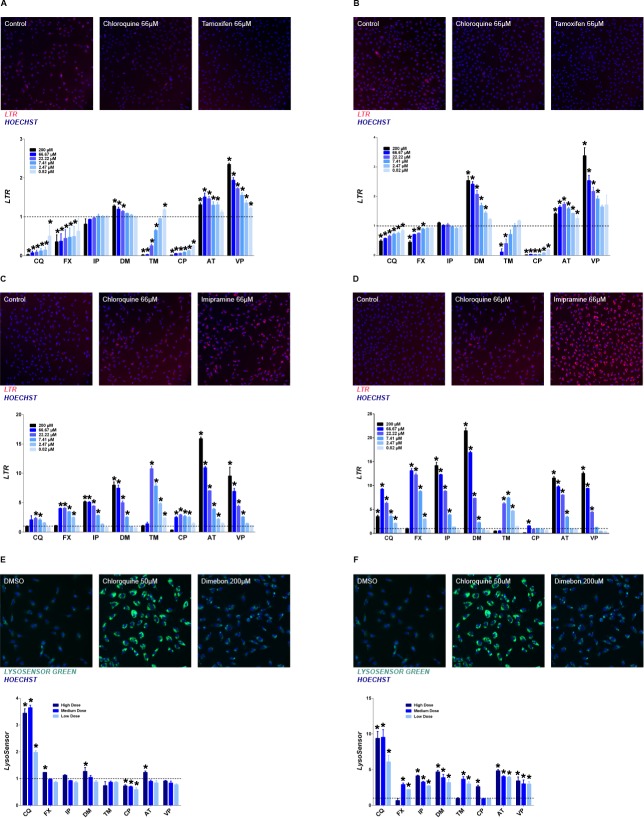
Effect of lysosomotropic drugs on lysosome volume and pH. Representative images for LTR after 30 mins (A), 1 hr (B), 4 hrs (C) or 24 hrs (D) and lysoSensor green after 4 hrs (E) or 24 hrs (F) of drug treatment. Quantification data in each panel is presented as fold change of the total signal intensity compared to control (mean ± SD), * p ≤ 0.05. Tested drugs include chloroquine (CQ), fluoxetine (FX), imipramine (IP), dimebon (DM), tamoxifen (TM), chloropromazine (CP), amitriptyline (AT), and verapamil (VP).

An increase in pH has been observed with lysosomotropic compounds [14] and hypothesized as a mechanism for lysosomal dysfunction. The fluorescent dye, LysoTracker Red^**®**^ DND-99 (LTR), which has been reported to accumulate in lysosomes by virtue of ionic trapping requiring acidic pH [15], was used to assess the lysosomal pH change with the drug treatment. However increase of LTR staining was also observed with lysosomotropic compounds treatment [12, 13]. In the current study multiple time points (30 mins, 1 hour, 4 hours and 24 hours) with a concentration range from 200 μM to 0.82 μM were evaluated after lysosomotropic compound treatment. After 30 mins of treatment chloroquine, fluoxetine, tamoxifen and chloropromazine was able to induce dose-dependent decrease of LTR staining as shown by the representative images and quantitative graph (Fig 1A). LTR change by imipramine is minimal at this time point whereas increase of LTR was observed for dimebon, amitriptyline and verapamil. After one hour (hr) of treatment, although the decrease of LTR were still observed for chloroquine, fluoxetine, tamoxifen and chloropromazine, compared to the response at 30 mins, restoration of LTR staining closer to control was noted for chloroquine, fluoxetine and tamoxifen. Similarly LTR staining was further increased for dimebon, amitriptyline and verapamil at one hr compared to response at 30 mins. After 4 hrs of exposure all test compounds increased LTR staining ranging from 2 to 15-fold compared to the vehicle control at their peak concentration (Fig 1C). A more pronounced increase was shown at the 24 hrs time point (Fig 1D) with LTR staining increases ranging from 7.3 to 21-fold. This data indicates that lysosomal pH increase (demonstrated by decrease of LTR staining at 30 mins and 1 hr) is a transient change and pH could be restored after extended exposure (demonstrated by increase of LTR staining at 4 hrs and 24 hrs). Additionally increase of LTR staining intensity could also indicates the increase of lysosome volume. For the rest of the studies, three concentrations were selected for each compound (Table 1)

To further confirm the pH normalization inside the lysosomes after 4 hrs and 24 hrs compound exposure the LysoSensor™ green probe was employed. The LysoSensor™ reagents exhibit a pH-dependent increase in fluorescence intensity upon acidification, in contrast to the LysoTracker® probes. Surprisingly, after 4 hrs drug treatment, no decrease in LysoSensor™ green signal was observed (Fig 1E), while chloroquine increased the staining intensity noticeably. The increase in LysoSensor™ green staining was more visible at 24 hrs (Fig 1F), ranging from 2.6 to 9.3 fold compared to control wells for all compounds tested. This further confirms the lack of pH increase by the drugs tested at 4 hrs and 24 hrs time points. Increased LysoSensor™ green staining intensity could be attributed to the increased lysosome volume.

### Lysosomal activation

**Fig 2:**
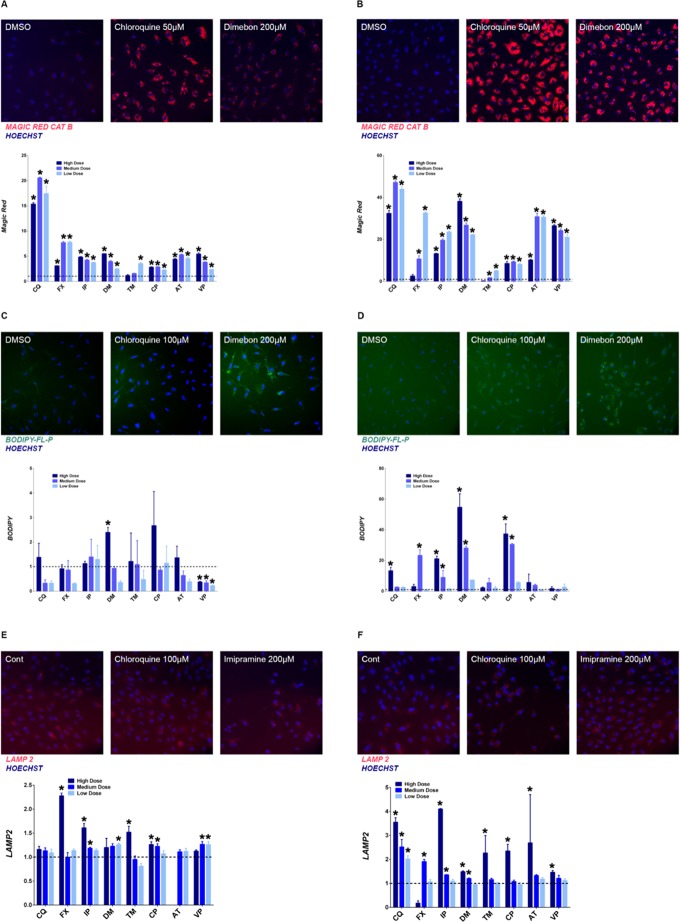
Increase of cathepsin B and D activity, and LAMP2 staining. Representative images for Cathepsin B activity (magic red staining) after 4 hrs (A) or 24 hrs (B), Cathepsin D activity (Bodipy-FL-P) after 4 hrs (C) or 24 hrs (D) and LAMP2 staining after 4 hrs (E) or 24 hrs (F) of drug treatment. Quantification data in each panel was presented as fold change of the total signal intensity compared to control (mean ± SD), * p ≤ 0.05. Tested drugs include chloroquine (CQ), fluoxetine (FX), imipramine (IP), dimebon (DM), tamoxifen (TM), chloropromazine (CP), amitriptyline (AT), and verapamil (VP).

To further assess lysosomal activity, the enzyme activities of lysosomal cathepsin B and D were measured. A cathepsin B-specific substrate (Magic Red fluorogenic) was used to evaluate cathepsin B activity. As shown in Fig 2A and 2B, an increase in intensity of Magic Red was observed at both 4 hrs and 24 hrs, with more pronounced staining at the later time point. This increase was also triggered by multiple concentrations of all selected compounds. Cathepsin D activation in lysosomes was measured using Bodipy FL-pepstatin A. An increase in cathepsin D was only observed at the high concentration of dimebon and chloropromazine at 4 hrs (Fig 2C), however by 24 hrs, all tested drugs increased cathepsin D activity at multiple concentrations (Fig 2D)

Lysosomal-associated membrane protein-2 (LAMP-2) constitutes a significant fraction of the total lysosomal membrane protein and thus the effect of drug treatment on LAMP-2 abundance was measured. Although the high concentration of fluoxetine, imipramine, and Tamoxifen were the only drugs to elicit a greater than 50% increase in LAMP2 staining at 4 hrs (Fig 2E), all drugs induced a 50% or greater increase in LAMP2 staining at one or more concentrations (Fig 2F) by the 24 hrs mark. The dose-dependent relationship is more noticeable for certain drugs (e.g. chloroquine and imipramine).

### Lysosomal biogenesis

**Fig 3:**
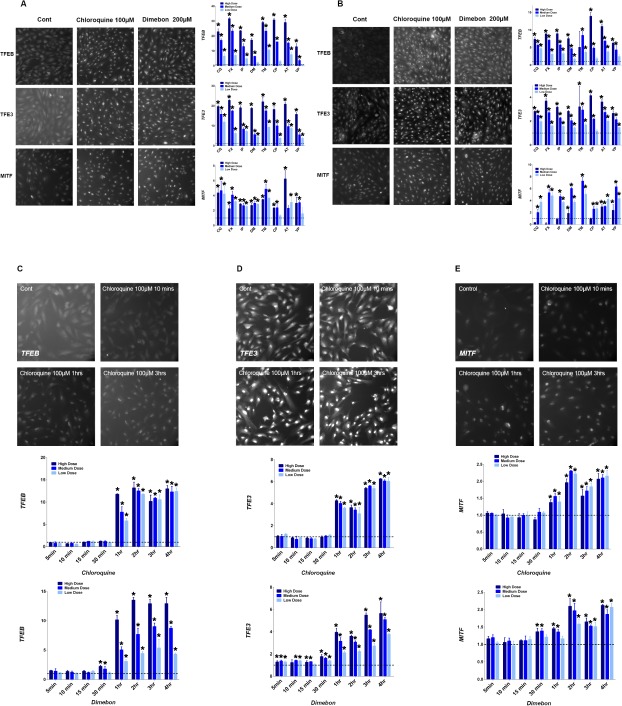
Increase of nuclear translocation of TFEB, TFE3 and MITF. Representative images for nuclear translocation of TFEB, TFE3 and MITF after 4 hrs (A) or 24 hrs (B) of drug treatment and for the time course of the nuclear translocation of TFEB (C), TFE3 (D) and MITF (E). Quantification data in each panel was presented as fold change of total signal intensity inside the nucleus compared to control (mean ± SD), * p ≤ 0.05. Tested drugs include chloroquine (CQ), fluoxetine (FX), imipramine (IP), dimebon (DM), tamoxifen (TM), chloropromazine (CP), amitriptyline (AT), and verapamil (VP).

Given the clear lysosomal activation demonstrated by increased lysosomal volume and cathepsin activity, lysosomal biogenesis induced by the compound treatment was investigated. A group of basic helix-loop-helix leucine zipper transcription factors including TFEB, TFE3, and MITF have been shown to play critical roles in lysosomal biogenesis [8, 9, 16]. To test the role of these transcription factors in drug-induced lysosomal biogenesis, immunofluorenscent staining was used to evaluate their location upon drug treatment. In the control samples, nuclear staining of TFEB, TFE3, and MITF was quite minimal. A striking dose-dependent increase in nuclear staining of TFEB, TFE3, and MITF was observed following 4 hrs (Fig 3A) and 24 hrs (Fig 3B) of drug treatment, indicating drug-induced translocation.

To further understand the kinetics of the cellular response to the tested drugs, a time course of the nuclear translocation of TFEB, TFE3, and MITF was conducted with chloroquine and dimebon. TFEB nuclear translocation increased abruptly after 1 hr of treatment with chloroquine. For dimebon, the increase of TFEB was observed as early as 30 mins, but was more pronounced by 1 hr (Fig 3C). In addition, the increase in TFEB nuclear translocation at 30 mins to the 4 hr time point was dose-dependent. The time course for MITF mirrored that of TFEB (Fig 3D and 3E). Although the change is marginal, statistically significant increase of TFE3 was observed for dimebon even after 5 mins of treatment. The prompt (30 mins to 1 hr) and enduring (tested up to 24 hrs) nuclear translocation of TFEB, TFE3, and MITF is consistent with the lysosomal activation shown previously.

**Fig 4:**
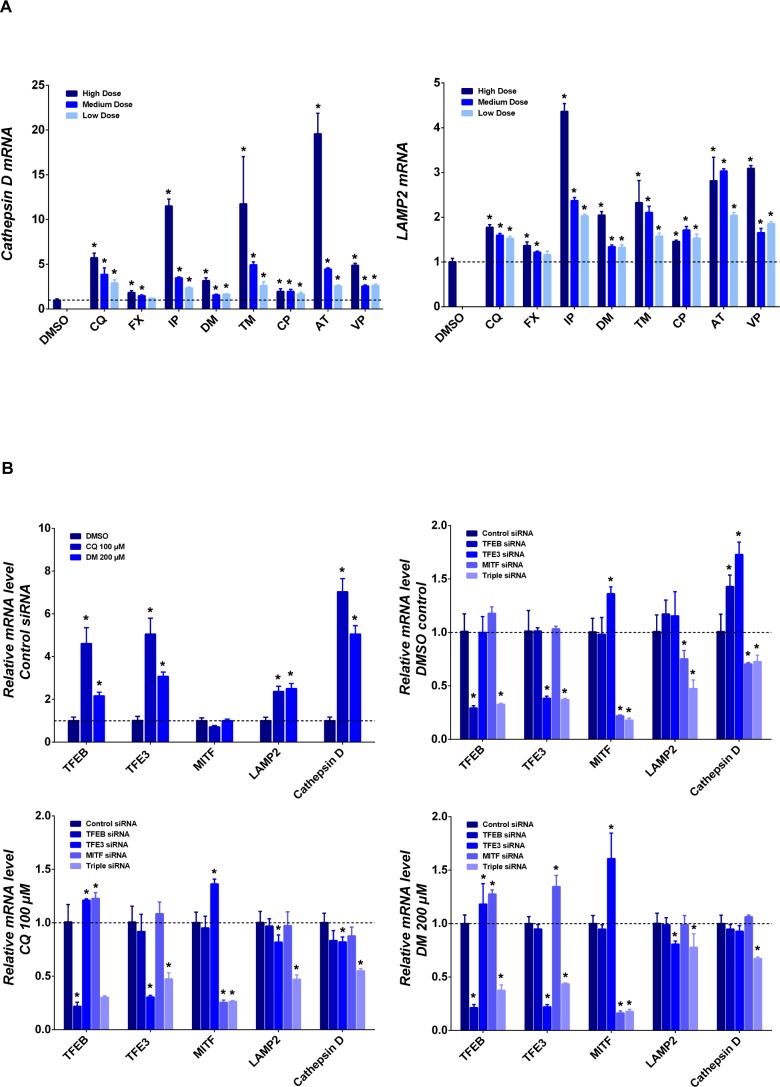
TFEB, TFE3 and MITF promote lysosome biogenesis in response to lysosomotropic Drugs. Relative mRNA expression of cathepsin D and LAMP2 was measured using RT-PCR (A). Quantification data in each panel was presented as fold change of mRNA expression in the treatment group compared to control (mean ± SD), * p ≤ 0.05. Tested drugs include chloroquine (CQ), fluoxetine (FX), imipramine (IP), dimebon (DM), tamoxifen (TM), chloropromazine (CP), amitriptyline (AT), and verapamil (VP); Relative mRNA expression of cathepsin D, LAMP2, TFEB, TFE3 and MITF was measured using RT-PCR with TFEB, TFE3 and MITF knockdown and selected drugs (B), Quantification data is presented as fold change of mRNA expression either in the treatment group compared to DMSO control (mean ± SD) or under knockdown condition compared to control siRNA treatment (mean ± SD), * p ≤ 0.05.

To further confirm the occurrence of lysosomal biogenesis, mRNA expression of cathepsin D and LAMP2 was assessed. An increase in mRNA level of both cathepsin D and LAMP2 was observed at multiple concentrations of all tested drugs (Fig 4A). Chloroquine (100 μM) and dimebon (200 μM) were used in the following siRNA knockdown experiments. Interestingly, mRNA expression of both TFEB and TFE3 were upregulated by both chloroquine and dimebon along with LAMP2 and cathepsin D (Fig 4B). MITF expression was not changed significantly at the 24 hrs time point.

Individual siRNAs for TFEB, TFE3, MITF and combination of all three siRNAs (triple siRNA) were used in the knockdown experiments to corroborate the role of these transcription factors (TFEB, TFE3 and MITF) on the transcriptional modulation of cathepsin D and LAMP2. Individual siRNAs specifically decreased mRNA levels of respective targeted transcription factors (Fig 4B) in the DMSO control group, and chloroquine (100 μM) and dimebon (200 μM) treatment groups. The triple siRNA also efficiently knocked down all three transcription factors. In the DMSO control, a decrease in mRNA level of LAMP2 and cathepsin D was only observed with MITF siRNA and the triple siRNA treatment, suggesting all three transcription factors, especially MITF, play a significant role on the basal expression level of LAMP2 and cathepsin D. In the chloroquine and dimebon treatment groups, pronounced mRNA decreases of cathepsin D and LAMP2 were only observed with the triple siRNA treatment, indicating that all three transcription factors are involved in the upregulation of the lysosomal biogenesis under the lysosomal stress condition. TFE3 single knockdown also decreased LAMP2 expression for both chloroquine and dimebon treatment.

### Decreased mTORC1 signaling by lysosomotropic compounds

**Fig 5:**
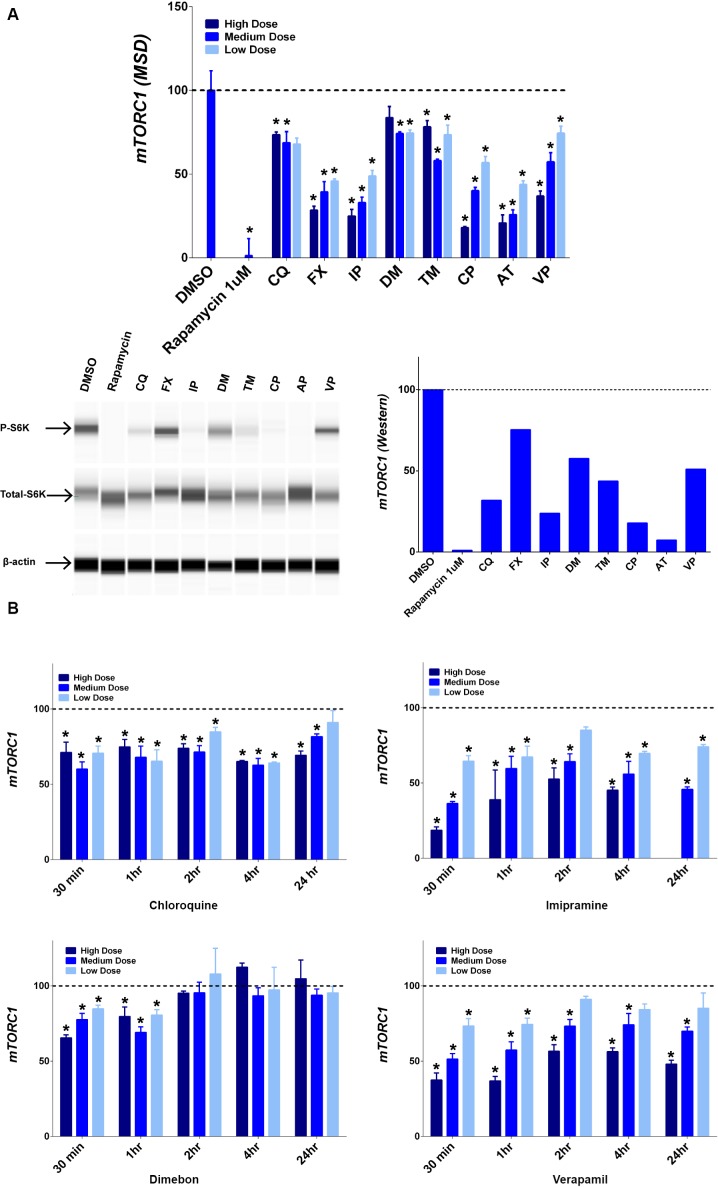
Decreased mTORC1 activity. mTORC1 activity was measured by the relative proportion of phosphorylated S6K (p-S6K/total S6K) using MSD technology (top panel) and Wes automated Western blotting system (bottom panel) for each drug (A). Time course of mTORC1 activity using MSD technology with selected drugs was shown (B). Quantification data is presented as percentage of mTORC1 activity compared to control (mean ± SD), * p ≤ 0.05. Tested drugs include chloroquine (CQ), fluoxetine (FX), imipramine (IP), dimebon (DM), tamoxifen (TM), chloropromazine (CP), amitriptyline (AT), and verapamil (VP).

Because mTORC1 phosphorylates and inhibits the transcriptional activity of TFEB, MITF, and TFE3 [9, 17], we tested whether mTORC1 activity was regulated by the selected drugs. mTORC1 activity was first evaluated using MSD ELISA method and all selected drug treatments decreased mTORC1 activity after 1 hr of treatment (Fig 5A, top panel), with certain drugs exhibiting dose-dependent decreases (e.g., fluoxetine & imipramine). The activity of mTORC1 was further analyzed using simple Western size-based capillary electrophoresis system and β actin was probed as a loading control. The positive control rapamycin (1μM) and all tested drugs (medium dose) diminished the mTORC1 activity after 1 hr treatment (Fig 5A, bottom panel), which confirms the finding with the MSD method.

mTORC1 activity was also evaluated at different time points after compound treatment to understand the kinetic change of mTORC1 activity using MSD technology. As shown in Fig 5B, chloroquine, imipramine, and verapamil all induced a persistent mTORC1 activity reduction up to 24 hrs. Only dimebon showed a transient reduction at 30 mins and 1 hr.

### Calcium signaling involvement in the nuclear translocation of TFEB and TFE3

**Fig 6:**
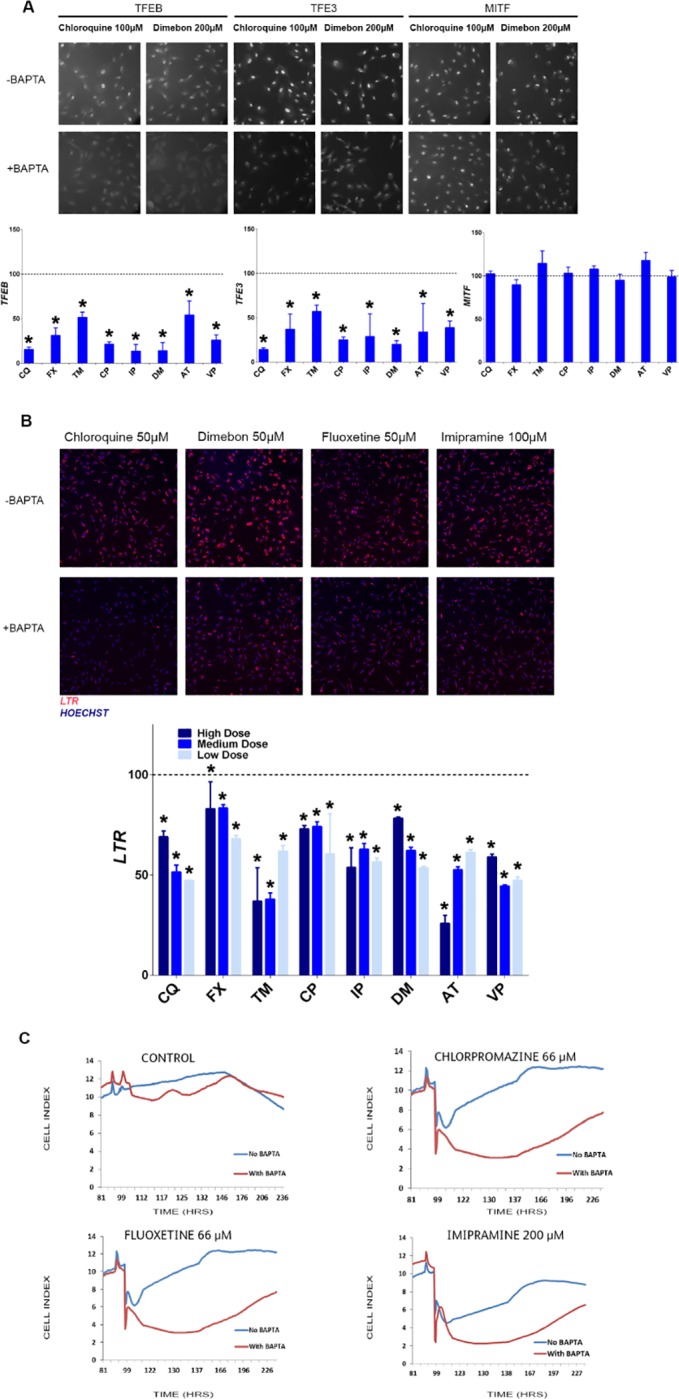
Involvement of calcium in the nuclear translocation of TFEB, TFE3 and MITF, and lysosomal activation. Representative images for nuclear translocation of TFEB, TFE3 and MITF (A) and LTR staining (B) with and without BAPTA co-treatment. The graphs represent the percentage of total signal intensity with BAPTA co-treatment compared to corresponding drug treatment alone (mean ± SD), * p ≤ 0.05. Kinetic cytotoxicity profile with and without BAPTA co-treatment (C). Tested drugs include chloroquine (CQ), fluoxetine (FX), imipramine (IP), dimebon (DM), tamoxifen (TM), chloropromazine (CP), amitriptyline (AT), and verapamil (VP).

Lysosomes have the ability to store calcium and participate in calcium signaling [18]. Lysosomal calcium signaling has been shown to affect calcineurin phosphatase activity toward its substrates TFEB and TFE3 [7, 19]. Furthermore, the accumulation of basic liphophilic drugs can cause the release of calcium from lysosomal stores [20]. To determine the role of calcium in nuclear translocation of the TFEB, TFE3, and MITF, a cell-permeant, highly selective Ca2+ chelator (BAPTA-AM) was used. As shown in Fig 6A, BAPTA pretreatment (5 μM for 1 hr.) decreased the drug-induced nuclear translocation of TFEB and TFE3, but not MITF. Additionally, BAPTA pretreatment also decreased the drug-induced LTR staining of all drugs and all concentrations tested (Fig 6B). This data indicates that calcium is involved in the nuclear translocation of TFEB and TFE3 under the lysosomal stress induced by the tested drugs and that BAPTA is sufficient to, at least partially, block the lysosomal changes. The effect of BAPTA on the kinetic cytotoxicity of selected drugs was assessed using impedance technology. Although BAPTA alone is slightly toxic to the cells at early time points (Fig 6C), BAPTA co-treatment with selected drugs caused synergistic cytotoxicity effects. This indicates that the lysosomal biogenesis triggered by the TFEB and TFE3 nuclear translocation has a protective role in the cells.

### Drug-induced lysosomal dysfunction

**Fig 7:**
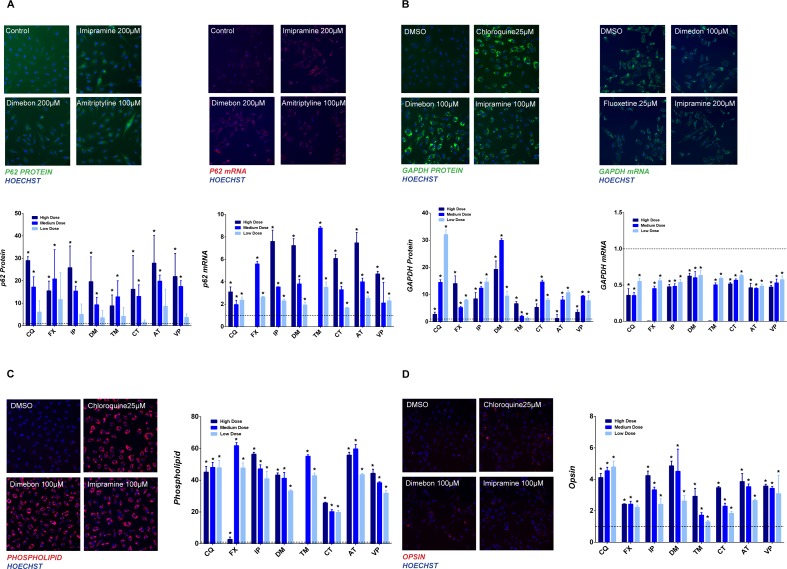
Decreased lysosomal function. Effect of drug treatment on the abundance of p62 protein and mRNA (A); GAPDH protein and mRNA (B); phospholipid (C); and opsin (D). Quantification data in each panel is presented as fold change of total signal intensity compared to control (mean ± SD), * p ≤ 0.05. Tested drugs include chloroquine (CQ), fluoxetine (FX), imipramine (IP), dimebon (DM), tamoxifen (TM), chloropromazine (CP), amitriptyline (AT), and verapamil (VP).

Given that the pH was not decreased and lysosomal biogenesis was activated by the lysosomotropic drugs, an array of different endpoints were employed to appraise the functional change of lysosomes induced by the tested drugs. All endpoints involve intracellular or extracellular substrates that require functional lysosomes for degradation. SQSTM1/p62 (hereafter p62) is a substrate delivered to lysosomes by the autophagy process for degradation and has been used as a marker to monitor autophagy flux [21]. At 24 hrs, all tested drugs increase p62 protein abundance in a dose dependent manner (Fig 7A left panel). A similar result was also observed when p62 was quantified using MSD methodology (data not shown). Interestingly, the mRNA level of p62 was also increased in a dose dependent manner as shown by the images and quantified data (Fig 7A right panel).

GAPDH (Glyceraldehyde 3-phosphate dehydrogenase) is a well-known substrate for chaperon-mediated autophagy and ultimately degraded by lysosomes [22, 23]. After 24 hrs of exposure, a drastic increase of GAPDH protein was observed shown by the representative images and quantified data (Fig 7B left panel). The test drugs induced a range of 6-30-fold increase of GAPDH staining at the concentration with peak response. In contrast to p62, the mRNA level of GAPDH decreased noticeably by visual observation and quantified data (Fig 7B right panel).

Lysosomal digestion of membranes is essential for cellular membrane homeostasis [24], and the most abundant class of lipid molecule found in cell membranes is the phospholipid. Therefore, phospholipid abundance was evaluated as a surrogate marker for lysosomal function. Remarkable increases in phospholipid were observed by visual inspection and quantified data (Fig 7C). A 25-fold or greater increase in phospholipid was triggered by all tested drugs.

In order to assess the degradation of extracellular material, photoreceptor outer segment was added to cells in the presence of drug treatment and opsin staining was used to monitor the degradation. Compared to the control, all tested drugs increased the opsin staining (Fig 7D), indicating blockage of photoreceptor degradation. Decreased degradation of the intracellular and extracellular substrates by lysosomotropic drugs suggests that the lysosomal functions are still perturbed despite the signal for increased lysosomal biogenesis and that the lysosome activation is likely a compensatory change aiming at reversing the lysosomal function deficiency.

## Discussion

The current studies aimed to characterize the impact of lysosomotropic compounds on the lysosomal pathway and how cells respond to this stress. Here we reported lysosomal activation (increase of LTR, LysoSensor green, cathepsin activity and LAMP2) by all tested compounds. Indeed, nuclear translocation of multiple transcriptional factors for lysosomal biogenesis including TFEB, TFE3, and MITF were prompted by all tested drugs. Chloroquine, the classic lysosomotropic compound, has been shown to trigger TFEB and TFE3 nuclear translocation due to lysosomal stress [9, 17]. The basic lipophilic nature of the other test drugs also makes them lysosomotropic, which triggers lysosomal stress. The increase in nuclear translocation of transcriptional factors for lysosomal biogenesis could simply be the adaptive response to lysosomal stress. In our studies we also identified the general mechanism for nuclear translocation of TFEB, TFE3 and MITF where reduction of mTORC1 activity and calcium participated in the signaling leading to the nuclear translocation of those transcription factors.

Lysosomotropic compounds are typically believed to increase lysosomal pH and consequently, decreased lysosomal enzyme activity. Previously, We did observe increased pH evident by decreased LTR staining following 4 hr drug treatment in H9C2 cells [14]. However, LTR increases have been observed with multiple other cell lines following 24 hrs treatment with lysosomotropic compounds [12, 13]. With ARPE19 cells, pH increase was rather transient and only observed at 30 mins and 1 hr exposure for Chloroquine, fluoxetine, tamoxifen, chloropromazine. By 4 hrs, all compounds increase LTR staining. Since LTR requires low pH to accumulate inside acidic organelles, an increase in LTR staining at 4 hrs and 24 hrs not only suggest that pH has been restored, but also is indicative of an increase in lysosome volume. In addition, lysosensor green data (Fig 1) supports the pH restoration at 4 hrs and 24 hrs, and thelack of a decrease in cathepsin activity further confirms the optimal conditions for lysosomal enzyme digestion.

Increased lysosome volume (LTR staining) and LAMP2 staining denotes the occurrence of lysosome biogenesis with the treatment of lysosomotropic drugs. Indeed, translocation of multiple transcription factors for lysosomal biogenesis, including TFEB, TFE3, and MITF, were triggered by all selected compounds (Fig 3). Increases in mRNA expression of cathepsin D and LAMP2 (Fig 4) further corroborate the notion of lysosomal biogenesis. Knockdown of those transcription factors decreased the mRNA level of LAMP2 and cathepsin D with compound treatment, which substantiates the role of TFEB, TFE3 and MITF mediating the lysosomal biogenesis triggered by lysosomotropic drugs. The more pronounced effect on LAMP2 and cathepsin D mRNA expression with triple transcription factor knock down compared to single transcription factor knockdown implies the redundancy among members of the MiTF/TFE family.

ARPE cells represent retinal epithelial cells, which functions as one of the most active phagocytic cell group and certainly require a fast lysosome response. Indeed as shown by the time course data (Fig 3), translocation of transcription factors for lysosomal biogenesis including TFEB, TFE3, and MITF started to increase substantially within 1hr following compound treatment. For the lysosomal pH change, kinetics including how much, how fast a compound sequester inside lysosome lumen and how efficiently the cells/lysosomes respond to the compound sequestration could dictate if the pH increase can be observed and the duration of the pH increase. For instance H9C2, which is derived from embryonic rat heart tissue, might not respond to lysosomal stress as promptly as ARPE19, leading to pH increase at 4 hrs (add reference) in comparison to ARPE19 with normalized lysosomal pH after lysosomotropic compound treatment.

The increase of cathepsin activity not only confirms the optimal conditions for lysosomal enzyme digestion but provides further evidence of lysosomal biogenesis. Although nuclear translocation of multiple transcription factors for lysosome biogenesis seems to be sufficient to maintain the pH and cathepsin activities, lysosomal dysfunction was still observed for all the tested drugs. Not only was there an accumulation of intracellular substrates, including p62, GAPDH, and phospholipid, but also extracellular substrate degradation was blocked (i.e. increase of opsin staining). The paradoxical findings indicate that the lysosomal biogenesis was not sufficient to recover full lysosomal function and was likely an adaptive response tolysosomal stress. Interestingly similar phenomena were also observed in lysosomal storage diseases, where lysosomal adaptation, including nuclear translocation of transcription factors for lysosomal biogenesis, and increase of LTR and lysosomal enzyme activities took place [5, 25]. However, despite lysosomal biogenesis induction, lysosomal functions are evidently perturbed in lysosomal storage diseases. Additional investigation is warranted to further understand the mechanism of lysosome dysfunction.

The concept of lysosomal adaptation is just starting to unravel. Rather than inert organelles, lysosomes have been shown to respond to intra- and extracellular cues including starvation, ER stress, and mitophagy [7, 9, 10, 26]. It is well established that both TFEB and TFE3 participate in cellular adaptation to starvation [9, 10]. When nutrients are scarce, these transcription factors are activated to promote lysosomal biogenesis and autophagy induction to adjust cellular metabolism. Recently, both TFEB and TFE3 have been shown to be part of the integrated ER stress response [7], and nuclear translocation of both transcription factors was also observed during Parkin-mediated mitophagy [26]. Translocation of TFEB and TFE3 to the nucleus following lysosomotropic compound treatment is fast (30 min to 1 hr) and kinetically similar to the starvation condition (< 30 mins), while TFEB nuclear translocation by mitophagy is slightly slower, 4 hrs after treatment. In contrast, activation of TFEB and TFE3 took place after 12 hr or longer of ER stress [7]. It is also noteworthy that the increase mRNA expression of TFEB and TFE3, but not MITF, was associated with lysosomotropic drug treatment. This result corresponds to the previous report that the TFEB promoter contains multiple CLEAR elements to which TFEB binds to further induce TFEB expression under starvation conditions [27]. Although there is no current direct evidence that TFE3 carries similar CLEAR elements for transcription regulation, our data presented here supports the possibility that TFE3 might employ a similar mechanism for positive feedback. This autoregulatory loop allows cells to rapidly respond to stress (e.g. starvation and lysosomal stress) and maintain a sustained response via positive feedback. Transcription of MITF does not seem to be regulated by both chloroquine and dimebon, which suggest a different regulatory mechanism for that lysosomoal biogeneic transcription factor.

Rapid mTORC1 inactivation was noted in the lysosomal stress condition, which was also observed during nutrition deprivation and mitophagy. Conversely, the activity of mTORC1 was not significantly reduced by ER stress. In contrast to nutrition deprivation where reduction of mTORC1 activity is transient [28], the compounds in our study triggered a more persistent decrease of mTORC1 activity (Fig 4), which could be ascribed to impaired lysosomal functions observed for those compounds (Fig 6). Indeed the lysosomal inhibitor leupeptin abolishes reactivation of mTOR during starvation process [28]. Likely the rapid mTORC1 inactivation observed for starvation and lysosomal stress could account for the faster TFEB and TFE3 nuclear translocation in comparison to the longer response time observed during ER stress.

Recently, lysosomal calcium release has been shown to play a crucial role in TFEB nuclear translocation in response to starvation [19]. Accumulation of lysosomotropic compounds can also lead to release of the lysosomal calcium stores by causing the breakdown the lysosomal membrane [20, 29]. Blocking the calcium signal using BAPTA/AM not only decreased the nuclear translocation of TFEB and TFE3, but also decreased LTR staining, indicating a role for calcium in the lysosomal biogenesis in response to lysosomal stress. Calcium modulation appears as a shared pathway for lysosomal biogenic response under various stress conditions such as starvation, ER stress and lysosomal stress [7, 19].

Interestingly, nuclear translocation of MITF does not appear to be impacted by the calcium blockage. Recently Wnt signaling has been shown to play a role in the MITF nuclear translocation [16], which could provide an alternative pathway to regulate MITF. Activation of MITF was observed under lysosomal stress and mitophagy initiation [26]. It would be of interest to determine if MITF is involved in the response to the other stresses (e.g., starvation and ER stress). Under all the stress conditions (starvation, ER stress, mitophagy, and lysosomal stress), multiple members from Mit/TFE family are activated and translocate into nucleus. This phenomenon signifies that lysosomal adaptation is a critical process for cells to respond to various environment cues, and multiple transcription factors are compelled to cooperatively regulate the lysosomal adaptation. This adaptive change seems to be protective for the cells, as observed in Fig 6C, yet it was not sufficient to restore lysosomal dysfunction marked by the accumulation of intracellular and extracellular substrates.

Drug discovery efforts have sought to boost lysosomal functions and enhance autophagy with the goal of lowering the burden of toxic protein aggregates, and ultimately providing therapeutic benefit. Large scale screening for autophagy modulators has been carried out in various labs [30–33]. Chloroquine, a well-known lysosomotropic compound, is generally accepted as an autophagy inhibitor. However numerous basic lipophilic compounds including all test compounds (except chloroquine) in this study have been reported as autophagy enhancers (Table 1). Unexpectedly, no differential results were seen between chloroquine and reported autophagy activators, raising a critical issue related to autophagy enhancer screening and drawing into question if the reported autophagy activators are true enhancers.

Based on our data, these basic lipophilic compounds, including chloroquine, all trigger nuclear translocation of transcription factors involved in lysosomal biogenesis. Genes involved in the major steps of the autophagic pathways have been shown to be controlled by the same transcriptional programs [6]. Therefore, these compounds will activate multiple genes related to autophagy, thereby increasing autophagic flux, which could be interpreted as positive in the autophagic flux assay. Compounds that simply increase the upstream autophagic flux without mending downstream fusion and degradation may not provide much therapeutic benefit and should be evaluated cautiously as true autophagy enhancers. For instance, thapsigargin, once shown to increase autophagy flux and considered an autophagy enhancer, has recently been demonstrated to block the fusion of the autophagosome and lysosomes [34]. Lysosomotropic drug-induced protein accumulation of both p62 and GAPDH further favors the notion of autophagy inhibition since those proteins are substrates degraded by lysosomes via autophagy and chaperon-mediated autophagy pathways, respectively. Also noteworthy is the difference in direction of mRNA change for these substrate, with an increase in p62 but a decrease in GAPDH. It has been shown p62 is one of the target genes for transcription factor TFEB [6, 35]. Not surprisingly, the increase in the transcription of p62 is associated with lysosomal adaptive change. Therefore, the p62 protein level increase is not simply due to autophagy blockage and lysosomal dysfunction, but also due to the increase of transcriptional activity. P62 is commonly used as a marker to evaluate autophagy process, and both protein and mRNA levels should be evaluated for better data interpretation. In contrast, the increase in the GAPDH protein is associated with a decrease in mRNA level, possibly due to negative feedback as we observed with the mRNA down regulation of long-lived proteins with lysosomotropic compound treatment [36]. Therefore, GAPDH may be used as additional marker of differentiation between enhancement and inhibition of autophagy.

Overexpression of TFEB and TFE3 has been shown to promote the elimination of storage material and ameliorate the pathological findings of various lysosomal storage diseases [9, 37, 38]. Screening efforts have been put in place to seek small molecules that could promote the nuclear translocation of these transcriptional factors. Given the lysosomal adaptive change observed for the lysosomotropic compounds, further experiments are warranted to discern autophagy induction from the adaptive change.

In conclusion, we not only observed lysosomal adaptive change in response to lysosomal stress, but also identified the mechanism by which both mTORC1 and calcium regulate lysosomal biogenesis when cells attempt to overcome lysosomal stress. These data further support the dynamic role of lysosomes as a signaling hub responding to various environmental cues. The fact that multiple transcription factors (TFEB, TFE3, and MITF) with overlapping functions are involved in the process suggests the lysosomal adaptive changes are critical for cells. However, the existence of lysosomal dysfunction in parallel with lysosomal adaptive changes triggered by the lysosomal stress indicates that simply promoting upstream biogenesis may not be sufficient to correct lysosomal function impairment. Improving downstream events including fusion, trafficking, and degradation may also be required to fully restore the lysosomal pathways, reinstate lysosomal function, and ultimately provide therapeutic benefits for diseases involving lysosomal dysfunction.

## Materials and methods

### Reagents and cells lines

All compounds were purchased from Sigma Aldrich ^®^ (St. Louis, MO). All studies were conducted using human retinal pigment epithelial cell line APRE-19 (ATCC, Manassas, VA). This cell line has been used to study lysosomal change and phagocytosis perturbation in our laboratory [39]. ARPE-19 can actively uptake photoreceptor outer segment *in vitro*. It has been shown to have high level of endogenous TFE3 [9]. MITF has also been shown to play a role in RPE development and differentiation [40]. These cells were maintained in DMEM: F12 (Life Technologies, Carlsbad, CA) media supplemented with 10% (v/v) heat-inactivated FBS, 100 units/ml of Penicillin/Streptomycin, and 2 mM L-Glutamine (Thermo Fisher Scientific, Waltham, MA) at 37°C in a humidified incubator with 5% CO_2_.

### High content LysoTracker ® Red (LTR), LysoSensor Green, Magic Red™ cathepsin B, Bodipy FL-pepstatin A for cathepsin D and phospholipid staining

ARPE-19 cells were seeded in 96-well plates at 5000 cells/well and allowed to attach overnight. The final DMSO concentration was 0.5% in vehicle control wells and compound treated wells. After drug treatment cells were then stained with LysoTracker® Red probes (Life Technologies, Carlsbad, CA) containing 60 nM LTR and 5 μg/ml Hoechst 33342 (Life Technologies, Carlsbad, CA). LysoSensor™ Green DND-189 (Life Technologies, Carlsbad, CA) was diluted to 1μM concentration for staining. For cathepsin B staining, all cells were incubated with Magic Red Cathepsin B Substrate (Immunochemistry Technologies, Bloomington, MN) following manufacture’s protocol. For cathepsin D staining, Bodipy FL-pepstatin A was diluted to 1μM concentration for staining. HCS LipidTOX™ Red phospholipidosis detection reagent (Life Technologies, Carlsbad, CA) was used to detect accumulation of phospholipid following manufacture’s protocol. The resultant images (> 6 images or more/well) were automatically captured and quantified using Thermo Fisher Scientific ArrayScan XTI (Thermo Fisher Scientific, Waltham, MA). Quantification of fluorescence intensity was conducted using cell health profiling algorithm, and the result is calculated as fold increase over vehicle control.

### High content immunofluorescent staining

Protein levels of LAMP2, TFEB, TFE3, MITF, p62, GAPDH, and opsin were assessed using immunofluorescent staining. Seeding of the ARPE-19 cells was similar to that with LysoTracker® Red. Cells were fixed with 4% paraformaldehyde for 15 minutes. Primary antibody was added for 1 hr at room temperature after initial permeabilization and blocking step. Cells were washed twice in dPBS prior to the addition of secondary antibody, Dylight 488 goat anti-rabbit, or Dylight 550 goat anti-mouse and nuclear counterstain, Hoechst dye. Cells were visualized after several washes in PBS. Following antibodies were used: anti-LAMP2 (Abcam, cat. ab25631), -TFEB (Cell Signaling cat. 4240), -TFE3 (Sigma-Aldrich, cat. HPA023881),–MITF (Sigma-Aldrich, cat. HPA003259),-p62 (Cell Signaling cat. 7695) -GAPDH (Cell Signaling cat. 2118), and -opsin (Sigma-Aldrich, cat. O4886). The resultant images (> 6 images or more/well) were automatically captured and quantified using Thermo Fisher Scientific ArrayScan XTI (Thermo Fisher Scientific, Waltham, MA). Quantification of fluorescence intensity was conducted using cell health profiling algorithm, and the result is calculated as fold increase over vehicle control.

### Impedance measurement for cytotoxicity

The cytotoxicity with and without BAPTA treatment was monitored using the xCELLigence® MP, a real-time analyzer (RTCA) based on the assessment of impedance variation, which uses a microelectronic 96-well plate (E-plate; ACEA Biosciences, San Diego, CA). The E-plate has gold microelectrodes integrated into the bottom of the wells. The cell index (CI), a dimensionless parameter derived from a relative change in the measured electrical impedance, is generated to represent cell viability in a real-time plot. When cells are not present or adhered, CI is zero. When more cells are attached to the electrodes, the CI values increase progressively and proportionally.

### Quantitative estimation of mTORC1 activity and p62 protein by MSD electro-chemiluminescence

Quantitative levels of Phospho-p70S6K (Thr389), total p70S6K, and p62 were measured using Meso Scale Discovery (MSD, Rockville, MD) Multi-Spot electrochemiluminescence technology. Briefly, cells were lysed in lysis buffer provided with the MSD kit containing Tris buffer supplemented with protease inhibitors. Cell lysates were incubated on ice for 30 mins and centrifuged at 20,000 *g* for 15 mins at 4°C to pellet the cell debris. MSD Multi-Spot p70S6K, p62 96-well plates were blocked with 150 μl of blocking buffer for 60 min at room temperature (RT) and washed three times in Tris wash buffer. The plates were incubated with 25 μl of protein lysate on the plate shaker at RT. The wells were then incubated with 25 μl of respective detection antibody per well for 60 min on the shaker. Plates were then washed with Tris wash buffer three times, and 150 μl of read buffer was added. The plates were read using a MSD Sector Imager 6000. MTORC1 signaling is represented by the normalized Phospho-p70S6K to total p70S6K. MTORC1 activity in the treated well is calculated as percentage over vehicle control, and result for p62/SQSTM1 is calculated as fold increase over vehicle control.

### Wes automated Western blotting for mTORC1 activity

After incubation with compounds for 1 hour, ARP19 cells at 96 wells were lysed in buffer containing phosphatase and protease inhibitors. The final samples of 5 μl each were boiled 5 min, placed on ice for 5 min, briefly centrifuged and applied to proper wells. Lysates were analyzed in the Simple Western size-based capillary electrophoresis system (ProteinSimple Wes; ProteinSimple, San Jose, CA). The size-separated proteins were probed with antibodies specific for phospho-p70S6K (Thr389; Cell Signaling Technology, no. 9205, 1:50), total p70S6K (Cell Signaling Technology, no. 9202, 1:50), or β-actin (Sigma-Aldrich, no. A3854, 1:200), visualized using labeled secondary antibodies, and quantitated using the manufacturers’ software. For each lane, the intensity of the phospho-p70S6K was normalized to the intensity of the total-p70S6K.

### mRNA quantification using RT-PCR

After incubation with compounds for 24 hrs, ARP19 cells at 96 wells were washed once with phosphate-buffered saline and processed using TaqMan® Gene Expression Cells-to-CT™ Kit (Thermo Fisher, Waltham, MA) following manufacturer’s instruction. Briefly, lysis solution containing DNase I was added and the samples were incubated for 5 min at room temperature. Stop solution was added and the samples were incubated for an additional 2 mins at room temperature. RNA samples for the evaluation of transcription knockdown effects were prepared from ARPE19 cells treated with siRNA (40 nM final concentration for TFEB and MITF, and 80 nM final concentration of TFE3) (GE Dharmacon, Lafayette, CO) and lipofectamine (Thermofisher, Waltham, MA) for 48 hrs prior to 24 hrs lysosomotropic compound treatment. Then TaqMan® 1-Step qRT-PCR was performed using primers specific for cathepsin D, LAMP2, TFEB, TFE3, MITF and β-actin.

### mRNA quantification using QuantiGene ViewRNA FISH cell assay for microscopy

To quantify the mRNA expression of GAPDH and p62/SQSTM1, RNA ISH using QuantiGene® ViewRNA cell assay kit (Affymetrix, Santa Clara, CA) was conducted. The oligonucleotide probe was designed commercially using the human GAPDH (accession number NM_002046) and p62 (accession number NM_003900) sequence. ARPE19 cells 5000 cells/well were plated on Poly-L-Lysine Coated 96 well plates and cultured overnight. Twenty-four hrs after compound treatment, the cells were permeabilized with working detergent solution, and digested with protease at 1:4000 in PBS. The cells were then hybridized for 3 hrs at 40°C with a cocktail of custom-designed QuantiGene ViewRNA probes against human GAPDH (type 1 probe) and p62 (type 6 probe). Unhybridized probes were flushed out with wash buffer, and the hybridized probes were amplified with pre-amp hybridization for 30 mins at 40°C, followed by amp hybridization for 30 mins at 40°C. Label Probes (LP) targeting the GAPDH and p62 were added for 30mins at 40°C. Cells were washed with wash buffer and plates were stained with DAPI. The resultant images (> 6 images or more/well) were automatically captured and quantified using Thermo Fisher Scientific ArrayScan XTI (Thermo Fisher Scientific, Waltham, MA). Quantification of fluorescence intensity was conducted using cell health profiling algorithm, and the result is calculated as fold increase over vehicle control.

### Statistics analysis

Results are presented as the means and standard deviations of independent samples. Data were statistically evaluated using one-way ANOVA test, followed by Dunnett post hoc test for compassion of individual treatment group with respected DMSO control group. P values ≤ 0.05 were considered to be significant and marked with an asterisk in figures.
